# How about your peers? Cystic fibrosis questionnaire data from healthy children and adolescents

**DOI:** 10.1186/1471-2431-11-86

**Published:** 2011-10-11

**Authors:** Marijke M Tibosch, Coosje JJCM Sintnicolaas, Jeannette B Peters, Peter JFM Merkus, Jan-Bart L Yntema, Christianne M Verhaak, Jan H Vercoulen

**Affiliations:** 1 Department of Medical Psychology, Radboud University Nijmegen Medical Centre, Nijmegen, The Netherlands; 2 Department of Pediatric Pulmonology, Radboud University Nijmegen Medical Centre, Nijmegen, The Netherlands; 3 Department of Pulmonary Diseases, Radboud University Nijmegen Medical Centre, Nijmegen, The Netherlands

## Abstract

**Background:**

The Cystic Fibrosis Questionnaire (CFQ) is widely used in research as an instrument to measure quality of life in patients with cystic fibrosis (CF). In routine patient care however, measuring quality of life is still not implemented in guidelines. One of the reasons might be the lack of consensus on how to interpret CFQ scores of an individual patient, because appropriate reference data are lacking. The question which scores reflect normal functioning and which scores reflect clinically relevant problems is still unanswered. Moreover, there is no knowledge about how healthy children and adolescents report on their quality of life (on the CFQ). With regard to quality of life the effect of normal development should be taken into account, especially in childhood and adolescence. Therefore, it is important to gain more knowledge about how healthy children and adolescents report on their quality of life and if there are any difference in a healthy populations based on age or gender. Without these data we cannot adequately interpret the CFQ as a tool in clinical care to provide patient-tailored care. Therefore this study collected data of the CFQ in healthy children and adolescents with the aim to refer health status of CF youngsters to that of healthy peers.

**Methods:**

The CFQ was completed by 478 healthy Dutch children and adolescents (aged 6-20) in a cross-sectional study.

**Results:**

The majority of healthy children (over 65%) did not reach maximum scores on most domains of the CFQ. Median CFQ-scores of healthy children and adolescents ranged from 67 to 100 (on a scale of 0-100) on the different CFQ-domains. Significant differences in quality of life exist among healthy children and adolescents, and these depend on age and gender.

**Conclusions:**

Reference data of quality of life scores from a healthy population are essential for adequate interpretation of quality of life in young patients with CF. Clinicians should be aware that the perception of health-related quality of life is not as disease-specific as one might think and also relies on factors such as age, normal maturation and gender.

## Background

Cystic fibrosis (CF) is the most common autosomal-recessive disease in the white population [1]. CF is, despite advances in care and treatment modalities, still a life threatening disease, involving especially the pulmonary and gastrointestinal organs. CF poses a heavy burden on patients and their families involving daily complex and time-consuming treatments with medication and demands of a healthy life style with sufficient nutrition and exercise [2]. Fortunately, life expectancy of patients with CF has improved dramatically in the recent years due to early diagnosis and better treatment options [3]. With this increasing life expectancy, patients with CF face new issues such as disease self management, development towards personal autonomy and identity, questions concerning intimate relationships and family planning. It is now recognized that traditional measurements of the physiological status (e.g. lung function and body mass index) are inadequate to cover all aspects of the impact of CF on daily life and on the broader issues mentioned above [4]. Within this context, measuring health-related quality of life has received growing attention in clinical care especially in pediatric and adolescent care [5-7]. Disease specific instruments are preferred above general instruments to measure health-related quality of life in patients with CF because they are designed to assess the symptoms and areas of functioning that are most important for CF [4]. Although several studies highlight the importance of incorporating these patient reported outcomes in routine care, assessing health-related quality of life is not yet implemented in clinical guidelines. This is probably partly due to the lack of appropriate reference values.

The Cystic Fibrosis Questionnaire (CFQ) was developed in 1997 as a CF specific questionnaire to measure various domains of health-related quality of life of children, adolescents and adults [8]. This instrument is now available in 25 languages and it is the most widely known instrument to measure a broad range of health-related quality of life domains in CF, in research and in clinical care. Health-related quality of life is not stable in time, but varies due to different factors such as life events, maturation, and changes in environment, treatment, disease, and coping abilities. The CFQ is the only disease specific questionnaire for patients with CF with several age-appropriate versions, which make it possible to follow patients with CF throughout their life span.

To date, however, there is no consensus on how to interpret CFQ scores of an individual patient with CF for clinical purposes, as no formal empirically derived reference data are available. There is some evidence about the minimally clinically important difference for the CFQ, but in this research normal maturation in a healthy population was not taken into account [9]. In the Netherlands, only two small studies provide preliminary reference data for a small sample of Dutch CF-patients [10,11]. In the first study psychometric characteristics of the Dutch CFQ were assessed in 84 adolescents and adults with CF aged 14-46 years. In the second study 68 children with CF (mean age 11.3 years) participated. Internal consistency was acceptable for most domains of the CFQ (α = 0.43-0.92) and test-retest reliability was high for all domain scores (0.72-0.98). However, it is unknown which score range reflects normal functioning and which score range reflects clinically relevant problems. Normality cannot be defined by absence of complaints or functional impairment, because even healthy subjects may experience symptoms such as fatigue or suffer from anxiety or depressed feelings from time to time. With regard to quality of life the effect of normal development has to be taken into account. For example, decreased mood and worries about body image is common with increasing age, especially in adolescence. Therefore, changes over time in quality of life may be due to progression of the disease but may also be due to normal maturation. Therefore, it is important to gain more knowledge about how healthy children and adolescents report on their quality of life, and if there are any differences based on age or gender in a healthy population. Without these data we cannot adequately interpret the CFQ as a tool in clinical care to provide patient-tailored care. The aim of this study was therefore to collect data on the CFQ in a healthy population of children and adolescents and to increase knowledge about this important patient reported outcome instrument in the care for patients with cystic fibrosis. We hypothesized that even healthy children would not gain maximum scores on a disease-specific health-related quality of life instrument.

## Methods

### Participants

In 2008, children and adolescents who met the inclusion criteria were invited to participate in this study. Inclusion criteria were: no chronic illness, not being under treatment of a medical specialist, age between 6 and 20 years, and the capability to understand and read the Dutch language. Children were recruited from primary and secondary schools in the Eastern region of the Netherlands. All primary and secondary schools in the city Nijmegen (42) were invited to participate. Six schools agreed to participate and in these schools classes were selected at random. In secondary school classes were selected from low to high education levels.

### Measures

The Cystic Fibrosis Questionnaire (CFQ) is a widely used disease specific questionnaire which measures different domains of health-related quality of life [12-15]. The CFQ was originally developed in France and designed for CF-care and research goals [8]. Psychometric properties of the Dutch version of the CFQ were good according to Klijn et al.[10]. For the purpose of the present study three Dutch versions were used:

-CFQ 6-11: interviewer administered version for children between 6 and 11 years old consisting of 35 items divided into 8 domains (physical functioning, emotional functioning, social functioning, body image, eating disturbances, treatment burden, respiratory symptoms, digestive symptoms).

-CFQ 12-13: self-administered version for children between 12 and 13 years old consisting of the same items and domains of the 6-11 version.

-CFQ 14+: self-administered version for adolescents and adults consisting of 50 items divided into 12 domains: the eight domains mentioned above and an additional four domains (role functioning, vitality, health perception and weight).

Response choices included ratings on a 4-point Likert scale. These scores are standardized and range from 0 to 100 on every domain with higher scores corresponding to better health-related quality of life. For the purpose of this study the domain 'treatment burden' with only disease specific questions about medical treatment for CF was omitted. We chose to use the 'old' version of the CFQ instead of the new CFQ-Revised, because the new CFQ-Revised was not psychometrically tested in the Netherlands and there are only small textual changes compared to the 'old' version [10]. The three versions of the Dutch CFQ are added as additional files 1, 2 and 3.

### Procedure

Three primary schools and three secondary schools in the Eastern region of the Netherlands took part in this study. Formal approval from the local medical ethics committee (CMO Arnhem-Nijmegen) was obtained. Written informed consent was obtained according to the principles of the local ethical committee. Legal caregivers of children under 16 were asked permission as well as adolescents above 12 years old. Participating children and adolescents filled out the paper and pencil version of the CFQ individually during a lesson in school. Children between 6 and 11 had individually interviewer administered questionnaires completed according to the manual.

### Analyses

Mean, median, standard deviation (SD) and range were computed for each domain of the CFQ in this reference population. Kolmogorov-Smirnov tests were used to test whether the CFQ domain scores showed a normal distribution. Analyses were separated for the three age-versions (children aged 6-11, children aged 12-13, and adolescents 14 -20 years old). The Mann-Whitney test was used to assess potential differences between gender and different age-groups. Data were analyzed with SPSS version 14.0.

## Results

### Participants

478 schoolchildren and adolescents participated in this study (table 1). Children and adolescents were derived from a representative sample of all types of education, ranging from low to high education levels.

**Table 1:** Sociodemographic characteristics of the sample

Variable	Total (n = 478) n (%)	Boys (n = 181) n (%)	Girls (n = 297) n (%)
Age (years)			
Mean ± S.D. (range)	14.52 ± 3.16 (6-20)	13.72 ± 3.09 (6-20)	15.00 ± 3.10 (6-20)
Age category (years)			
Young children (aged 6-11 years)	60 (12.6%)	31 (52%)	29 (48%)
Elder children (aged 12-13 years)	120 (25.1%)	58 (48%)	62 (52%)
Adolescents (aged 14-20 years)	298 (62.3%)	92 (31%)	206 (69%)

### CFQ-scores of healthy children and adolescents

Table 2 shows descriptive characteristics for the CFQ domains in the three age groups. Median scores ranged from as low as 67 ('Vitality' in adolescents and 'Digestive symptoms' in young children aged 6-11 years) to maximum scores of 100 ('Body image' in children aged 6-13 years 'Eating disturbances', 'Role functioning', and 'Weight' in adolescents). The Kolmogorov-Smirnov test demonstrated that all domain scores on the different CFQ-domains were not equally distributed (all domains p < 0.05). In the age group 6-11 years no gender differences were found. In the children aged 12 and 13 years old differences were found between boys and girls on the domains 'Emotional functioning' and 'Digestive symptoms', where girls reported more problems than boys, but there was major overlap (Emotional functioning median score female = 79 and male = 83, p = 0.005; Digestive symptoms median score female = 67 and male = 100, p = 0.025). In the adolescent group, male adolescents reported better quality of life status on the following domains: 'Physical functioning', 'Health perception', 'Eating disturbances' and 'Emotional functioning' compared to female adolescents (Physical functioning median score female = 92 and male = 96, p = 0.000; Health perception median score female = 78 and male = 89, p < 0.001; Eating disturbances median score female = 100 and male = 100, p = 0.007; Emotional functioning median score female = 80 and male = 87, p = 0.003). These data illustrate the heterogeneity between the different domains of the CFQ in a healthy population.

**Table 2:** Descriptive analyses (mean, SD, range, median and % maximum scores) of CFQ domain scores in healthy children (n = 478)

Variable	Mean	SD	Range	Median	n maximum score (% maximum scores)
**Healthy young children (aged 6-11) (n = 60)**					
Age	8.93	1.58			
Physical functioning	89.07	14.27	22-100	94	19 (31.7)
Emotional functioning	83.27	12.09	54-100	88	5 (8.3)
Eating disturbances	90.83	11.31	56-100	95	30 (50)
Social functioning	89.65	12.91	48-100	95	23 (16.7)
Body image	95.78	8.37	67-100	100	44 (73.3)
Respiratory symptoms	88.70	11.55	50-100	92	15 (25)
Digestive symptoms	80.13	20.49	33-100	67	28 (46.7)
**Healthy elder children (aged 12-13) (n = 120)**					
Age	12.30	0.93			
Physical functioning	88.41	13.25	17-100	92	47 (39.2)
Emotional functioning	79.89	14.52	17-100	83	8 (6.7)
Eating disturbances	85.57	16.74	33-100	89	47 (39.2)
Social functioning	84.95	12.97	38-100	86	27 (22.5)
Body image	93.20	11.93	44-100	100	79 (65.8)
Respiratory symptoms	90.68	10.23	33-100	92	35 (29.2)
Digestive symptoms	82.37	18.70	33-100	84	60 (50)
**Healthy adolescents (aged 14-20) (n = 298)**					
Age	16.53	1.67			
Physical functioning	90.04	11.66	38-100	92	84 (28.2)
Vitality	63.33	13.80	33-100	67	1 (0.3)
Emotional functioning	78.20	14.37	7-100	80	15 (5)
Eating disturbances	93.52	12.03	11-100	100	193 (64.8)
Health perceptions	80.78	16.74	33-100	84	74 (24.8)
Social functioning	90.26	9.14	33-100	94	73 (24.5)
Body image	84.59	17.77	22-100	89	126 (42.3)
Role functioning	93.81	7.94	67-100	100	157 (52.7)
Weight	93.42	19.47	0-100	100	260 (87.2)
Respiratory symptoms	92.21	11.34	33-100	94	131 (44)
Digestive symptoms	89.98	13.82	0-100	89	144 (48.3)

**Figure 1:**
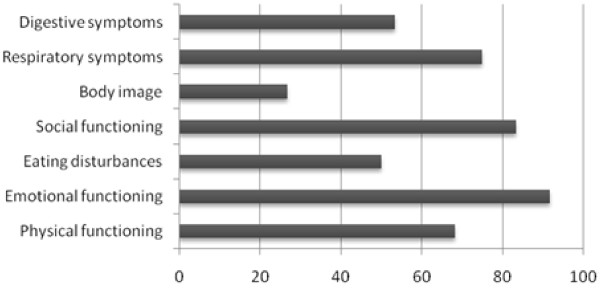
**Percentage of healthy children aged 6-11 years NOT reaching maximum scores on the different CFQ domains (n = 60)**.

**Figure 2:**
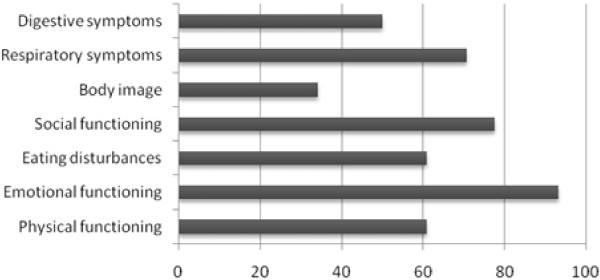
**Percentage of healthy children aged 12-13 years NOT reaching maximum scores on the different CFQ domains (n = 120)**.

**Figure 3:**
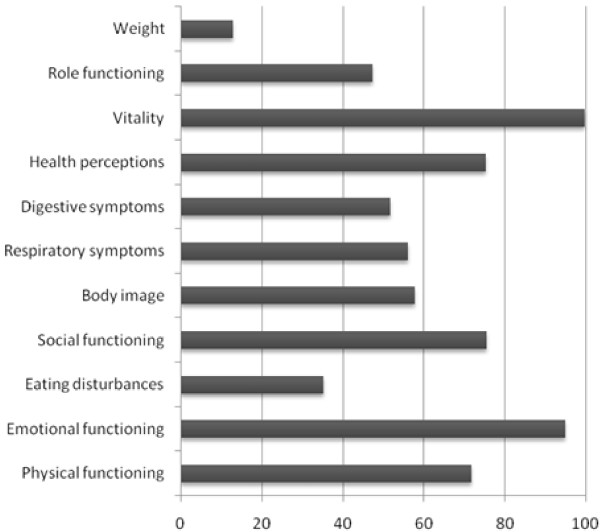
**Percentage of healthy adolescents aged 14-20 years NOT reaching maximum scores on the different CFQ domains (n = 298)**.

Figure 1, 2 and 3 demonstrate the percentage of healthy children for each age group who do not reach maximum scores on the different domains of the CFQ. There is not one domain of the CFQ in which all healthy children reach maximum scores.

**Figure 4:**
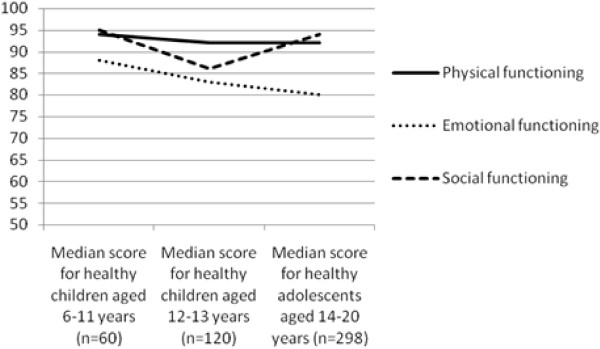
**Median scores for the three age groups on three domains of the CFQ (physical functioning, emotional functioning and social functioning)**. ^a ^Differences between physical functioning are not significant in the three age-groups^b ^Difference between emotional functioning is significant between healthy young children aged 6-11 years and healthy adolescents (p < 0.05, Mann-Withney)^c ^Difference between social functioning is significant between healthy children aged 6-11 years and healthy children aged 12-13 years (p < 0.01, Mann-Whitney), and between healthy young children aged 12-13 years and healthy adolescents (p < 0.01, Mann-Whitney).

**Figure 5:**
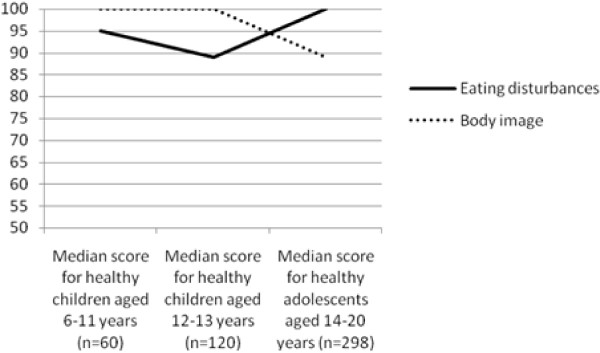
**Median scores for the three age groups on two domains of the CFQ (eating disturbances and body image)**. ^a ^Difference between body image is significant between healthy young children aged 6-11 years and healthy adolescents (p < 0.01, Mann-Whitney), and between healthy young children aged 12-13 years and adolescents (p < 0.01, Mann-Whitney)^b ^Difference between eating disturbance is significant between healthy young children aged 6-11 years and healthy adolescents (p < 0.05, Mann-Whitney), and between healthy young children aged 12-13 years and adolescents (p < 0.01, Mann-Whitney).

**Figure 6:**
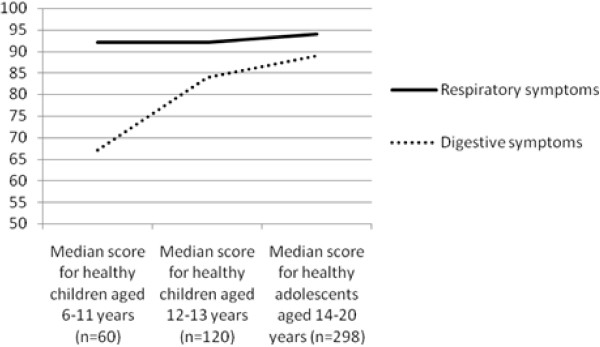
**Median scores for the three age groups on two domains of the CFQ (respiratory symptoms and digestive symptoms)**. ^a ^Difference between respiratory symptoms is significant between healthy young children aged 6-11 years and healthy adolescents (p < 0.01, Mann-Whitney), and between healthy young children aged 12-13 years and adolescents (p < 0.01, Mann-Whitney)^b ^Difference between digestive symptoms is significant between healthy young children aged 6-11 years and healthy adolescents (p < 0.01, Mann-Whitney), and between healthy young children aged 12-13 years and adolescents (p < 0.01, Mann-Whitney).

Figures 4, 5 and 6 illustrate the trend for median scores of the different CFQ domains for the three age groups. These figures demonstrate that CFQ-scores are different for the subsequent age groups. Some symptoms are more frequent in younger healthy children than in healthy adolescents (e.g. digestive symptoms) and some domains are decreasing when children grow older (e.g. adolescents report more problems on the domain 'Body image' than younger children).

## Discussion

### Principal findings

This study has four major outcomes. The first conclusion is that the majority of healthy children do not reach maximum scores on many domains of the CFQ. The percentage of healthy children and adolescents reaching maximum scores on the different CFQ-domains was remarkably low with an average of 34.8% over the different domains. For example, in the domain 'Vitality'' 99.7 percent of all healthy adolescents did experience feelings of tiredness and exhaustion to some degree. This finding is in agreement with the statement that normality cannot be defined as the absence of symptoms or functional impairment.

The second major finding is the heterogeneity of scores between different CFQ-domains in healthy subjects. Median scores on the different CFQ-domains varied between 67 and 100. For example, digestive symptoms (e.g. abdominal pain, flatulence, and diarrhea) are apparently quite common in young healthy children (median score = 67 on domain 'Digestive symptoms' in children aged 6-11 years old). In contrast, eating disturbances did not occur very often in this healthy age group (median score 95).

The third finding is that median scores on different domains of the CFQ differed according to age. As mentioned above, digestive symptoms occurred frequently in healthy young children aged 6-11 years old (median score 67), in contrast to the relative absence of digestive symptoms in the age group of healthy adolescents, in which digestive symptoms were quite rare (median score 89). Body image becomes more problematic when children grow into adolescence. This finding is in agreement with previous research in which most general quality of life scales (e.g. emotional functioning, physical and psychological wellbeing and self-perception) decrease from childhood into adolescence [16,17]. A community based study showed that abdominal pain is more frequently reported in childhood than in adolescence [18].

The last major finding is that there were some sex-related differences. In 6-11 year old healthy children there were no differences in any domain of the CFQ between boys and girls. In children aged 12-13 years old, girls reported more problems with emotional functioning and digestive symptoms, but there was major overlap. In the age-group 14-20 years, boys reported better quality of life on the domains: 'Physical functioning', 'Health perception', 'Eating disturbances' and 'Emotional functioning'. But also in these domains there was considerable overlap in scores. These findings are in agreement with observations that in general, female adolescents have a poorer perception of their own health and report more somatic problems than their male counterparts [17,18]. These data indicate that it is important to take into account gender in the evaluation of health-related quality of life.

### Methodological considerations and implications for future research

The major strength of our study is that, to our knowledge, this is the first study in which data of a large group of healthy children and adolescents was used to expand knowledge on a disease specific instrument for CF. These data help to improve the interpretation of health-related quality of life assessment in CF using the CFQ. However, some methodological issues need further comment. In the adolescent group we had a disproportional high rate of females in our study-sample, due to the particular schools which participated. This may have influenced our results, in some way. The difference in the domain eating disturbances between young children and adolescents may be even bigger. The difference between emotional functioning of young children aged 12-13 years and adolescents might be smaller in reality.

It is impossible to determine whether differences in CFQ scores between the three age groups are due to true age differences or whether differences in CFQ scores are caused by the fact that different, age-specific questionnaires and procedures were employed for each age group. In the youngest age group the CFQ was administered individually with an interviewer, whereas the older children and adolescents filled out the CFQ in the classroom without individual help. Because only Dutch children and adolescents were included, it is unknown whether our results are also representative for other countries. Future research should collect reference data from healthy adults and from a large clinical population of CF patients in all age groups in different countries. A longitudinal study would be needed to assess how health-related quality of life evolves over time in individuals.

Obviously, the CFQ can not be used as a quality of life measure for healthy children because it does not cover all aspects of quality of life in healthy children. It is especially designed for the evaluation of health-related quality of life of patients with CF. Therefore, the primary objective of this study is to optimize the interpretation of CFQ-scores in young patients with CF. The CFQ allows specific issues to be identified, addressed and monitored over time. However comparison against 'norms' is not always wanted. This new data allows us to see whether we are concerned about a CFQ score which is inherently 'normal' or not for the CF child's age and gender, but it must also be related to whether it is perceived as a problem regardless of whether it relates to the influence of the CF on the child or not.

### Consequences for patient care

This study demonstrates that the majority of healthy children and adolescents do not gain maximum scores on a disease specific health-related quality of life instrument and that there are many differences between the outcomes on the different domains and age groups. This is important information if we want to interpret the health-related quality of life for individual patients based on the CFQ for treatment purposes. In healthy adolescents aged 14-20 most subjects scored on the domain 'Vitality' under 70 (median: 67), indicating that many healthy adolescents report feelings of tiredness, lack of energy or exhaustion. Therefore, a 'Vitality' score of 70 in an adolescent with CF does not automatically indicate a relation to his or her disease, but it can also be related to other factors NOT related to CF. Similarly, in children with CF, a suboptimal CFQ-score in the domain 'Digestive symptoms', does not always implicate that these symptoms are due to their disease because recurrent abdominal pain is a common complaint in normal childhood and in many cases an underlying cause is not found [19]. However, a score of 70 in a young boy (aged 7 years) with CF on the domain 'Body image' is low compared to age-matched healthy controls, and deserves more CF-specific attention. This study indicates that for the clinical interpretation of health-related quality of life of an individual patient with CF it is essential to pay attention to contextual non-disease specific factors such as age, maturation and gender. Incorporating disease specific questionnaires that assess different domains of quality of life in routine CF care may improve our understanding of the impact of CF on daily life [7]. A better understanding of this impact can facilitate communication between health care providers and patients. In addition, more patient-tailored treatments may be developed. As previous research has shown, better communication can improve treatment adherence [20-22]. Improving treatment adherence can improve quality of life in a majority of CF-patients.

## Conclusion

The results of the present study provide us with a tool to optimize the interpretation of CFQ-scores in young patients with CF. Clinicians should be aware that the perception of health-related quality of life is not as disease-specific as one might think and also relies on factors such as age, maturation and gender. Health-related quality of life scores based on the CFQ of individuals with CF should be interpreted within the context of normal development. A careful interpretation of health-related quality of life helps to improve patient-tailored treatment.

## Competing interests

The authors declare that they have no competing interests

## Authors' contributions

MT performed the statistical analysis and wrote the manuscript. JHV was the main supervisor and designed the study together with CJS, who collected the data and drafted the manuscript. JBP guided the statistical analyses and study protocol. All other authors are involved in optimization of the study protocol, supervision of data collection, writing of the manuscript and critically revising it for important intellectual content. All authors read and approved the final manuscript and declare that they have nothing to declare.

## Pre-publication history

The pre-publication history for this paper can be accessed here:

http://www.biomedcentral.com/1471-2431/11/86/prepub

## Supplementary Material

Additional file 1 **Cystic Fibrosis Questionnaire 6-11 Dutch version**. Cystic Fibrosis Questionnaire for children aged 6-11 years (Dutch version). Click here for file

Additional file 2 **Cystic Fibrosis Questionnaire 12-13 Dutch version**. Cystic Fibrosis Questionnaire for children aged 12-13 years (Dutch version). Click here for file

Additional file 3 **Cystic Fibrosis Questionnaire 14+ Dutch version**. Cystic Fibrosis Questionnaire for children aged 14 and/or older, and adults (Dutch version). Click here for file
