# Supplementary material for: How about your peers? Cystic fibrosis questionnaire data from healthy children and adolescents
Source: BMC Pediatr. 2011 Oct 11;11:86. doi: 10.1186/1471-2431-11-86 (PMC3198681; doi:10.1186/1471-2431-11-86)
Supplement: Additional file 1 — Cystic Fibrosis Questionnaire 6-11 Dutch version. Cystic Fibrosis Questionnaire for children aged 6-11 years (Dutch version). [file 1471-2431-11-86-S1.PDF]

CFQ CYSTIC FIBROSIS VRAGENLIJST

Kinderen 6 t/m 11 jaar (Interviewer Format)

Deze vragenlijst is opgesteld voor gebruik door een interviewer. Gebruik deze versie alleen voor kinderen van 6 t/m 11 jaar. Gebruik voor oudere kinderen, die in staat lijken de vragen zelf te lezen en te beantwoorden, zoals 12- en 13-jarigen, deze vragenlijst in de versie voor zelf-rapportage.

Er zijn aanwijzingen voor de interviewer bij elke sectie van de vragenlijst. Aanwijzingen die u het kind moet *voorlezen* zijn aangegeven door aanhalingstekens. Aanwijzingen die u moet *opvolgen* zijn onderstreept en staan in cursief.

INTERVIEWER: *Stel de volgende vragen*

A. Wat is je geboortedatum?

Datum ☐☐ ☐☐ ☐☐

Dag Maand Jaar

B. Ben je een?

☐ Man ☐ Vrouw

C. Ben je in de **afgelopen 2 weken** normaal naar school geweest?

☐ Ja ☐ Nee

Zo nee, had dit iets te maken met je gezondheid?

☐ Ja ☐ Nee

D. In welke groep zit je nu?
(Als het zomer is, in welke groep heb je het afgelopen jaar gezeten?)

☐ groep 2 basisschool
☐ groep 3 basisschool
☐ groep 4 basisschool
☐ groep 5 basisschool
☐ groep 6 basisschool
☐ groep 7 basisschool
☐ groep 8 basisschool
☐ niet op school

In te vullen door testafnemer:

Datum ☐☐ ☐☐ ☐☐ (dag mnd jaar) Centrum ☐

1e letters voornaam ☐☐ Patiënt # ☐☐☐☐☐☐☐☐

1e letters achternaam ☐☐ Vanwege ☐

©2000 Quittner, Buu, Watrous en Davis

INTERVIEWER:

Lees het kind het volgende voor:

"Deze vragen zijn voor kinderen als jij die Cystic Fibrosis hebben. Je antwoorden zullen ons helpen te begrijpen wat de ziekte betekent en hoe je behandelingen jou helpen. Daarom zal het beantwoorden van deze vragen jou en andere kinderen net als jij in de toekomst helpen."

"Kies bij elke vraag die ik stel, één van de antwoorden op de kaarten die ik je zo laat zien."

Laat het kind de oranje kaart zien.

"Kijk naar deze kaart en lees met me mee wat er staat: **heel erg waar/ voor een groot deel waar/ een beetje waar/ helemaal niet waar"**

"Hier is een voorbeeld: als ik je vraag of olifanten kunnen vliegen, welke van de 4 antwoorden op de kaart zou je dan kiezen: **heel erg waar, voor een groot deel waar, een beetje waar, helemaal niet waar**?"

Laat het kind de blauwe kaart zien.

"Kijk nu naar deze kaart en lees met me mee wat er staat: **altijd / vaak / soms / nooit".**

"Hier is nog een voorbeeld: als ik je vraag of je naar de maan gaat, welk antwoord op de kaart zou je dan kiezen: **altijd, vaak, soms of nooit**?"

Laat het kind de oranje kaart zien.

"Nu ga ik je een aantal vragen stellen over je dagelijks leven"

"Vertel me of je vindt dat de volgende beweringen (dingen) die ik je voorlees **heel erg waar, voor een groot deel waar, een beetje waar, helemaal niet waar** zijn"

Kruis het hokje aan met het antwoord van het kind.

"In de afgelopen **2 weken:**"	Heel erg waar	Voor een groot deel waar	Een beetje waar	Helemaal niet waar
**1.** Kon je net zo snel lopen als andere kinderen	☐	☐	☐	☐
**2.** Kon je net zo snel traplopen als andere kinderen	☐	☐	☐	☐
**3.** Kon je rennen, springen en klimmen zoveel als je wilde	☐	☐	☐	☐
**4.** Kon je net zo snel en zo lang rennen als andere kinderen	☐	☐	☐	☐
**5.** Kon je meedoen met de sporten die je leuk vindt (bijvoorbeeld zwemmen, voetbal, dansen of andere)	☐	☐	☐	☐
**6.** Had je moeite zware dingen te dragen of te tillen zoals boeken, je schooltas of rugzak	☐	☐	☐	☐

Patiënt # ☐☐☐☐☐☐☐☐

©2000 Quittner, Buu, Watrous en Davis

INTERVIEWER: *Laat het kind de blauwe kaart zien.*

Kruis het hokje aan met het antwoord van het kind.

"En, in deze afgelopen **2 weken**, vertel me hoe vaak:"

	Altijd	Vaak	Soms	Nooit
**7.** Voelde je je moe	☐	☐	☐	☐
**8.** Voelde je je kwaad	☐	☐	☐	☐
**9.** Voelde je je mopperig	☐	☐	☐	☐
**10.** Maakte je je zorgen	☐	☐	☐	☐
**11.** Voelde je je verdrietig	☐	☐	☐	☐
**12.** Had je moeite in slaap te vallen	☐	☐	☐	☐
**13.** Had je enge dromen of nachtmerries	☐	☐	☐	☐
**14.** Voelde je je blij met jezelf	☐	☐	☐	☐
**15.** Had je moeite met eten	☐	☐	☐	☐
**16.** Moest je stoppen met spelen vanwege je behandelingen, zoals fysiotherapie, ademhalingsoefeningen, vernevelen, etc.	☐	☐	☐	☐
**17.** Moest je toch eten, terwijl je dat eigenlijk niet wilde	☐	☐	☐	☐

INTERVIEWER: *Laat het kind de oranje kaart zien.*

"Vertel me nu of je vindt dat de beweringen (dingen) die ik je voorlees **heel erg waar, voor een groot deel waar, een beetje waar** of **helemaal niet waar** zijn"

Kruis het hokje aan met het antwoord van het kind.

"In de afgelopen **2 weken:**"

	Heel erg waar	Voor een groot deel waar	Een beetje waar	Helemaal niet waar
**18.** Kon je al je behandelingen doen	☐	☐	☐	☐
**19.** Had je plezier in eten	☐	☐	☐	☐
**20.** Heb je veel met vriendjes / vriendinnetjes gespeeld	☐	☐	☐	☐
**21.** Ben je meer thuis gebleven dan je wilde	☐	☐	☐	☐

©2000 Quittner, Buu, Watrous en Davis

"In de afgelopen **2 weken:**"	Heel erg waar	Voor een groot deel waar	Een beetje waar	Helemaal niet waar
**22.** Voelde je je op je gemak om uit logeren te gaan	☐	☐	☐	☐
**23.** Voelde je je buitengesloten	☐	☐	☐	☐
**24.** Heb je vaak vriendjes/vriendinnetjes bij je thuis uitgenodigd	☐	☐	☐	☐
**25.** Ben je geplaagd door andere kinderen	☐	☐	☐	☐
**26.** Vond je het makkelijk om over je ziekte met anderen te praten (vrienden, klasgenootjes, onderwijzers, etc.)	☐	☐	☐	☐
**27.** Dacht je dat je te klein was	☐	☐	☐	☐
**28.** Dacht je dat je te mager was	☐	☐	☐	☐
**29.** Dacht je dat je lichamelijk anders was dan andere kinderen van jouw leeftijd	☐	☐	☐	☐
**30.** Vond je het vervelend om je behandelingen te doen	☐	☐	☐	☐

INTERVIEWER: *Laat het kind de blauwe kaart weer zien.*

Kruis het hokje aan met het antwoord van het kind.

"Vertel me hoe vaak in de afgelopen **2 weken**:"	Altijd	Vaak	Soms	Nooit
**31.** Hoestte je overdag	☐	☐	☐	☐
**32.** Werd je 's nachts wakker omdat je hoestte	☐	☐	☐	☐
**33.** Moest je slijm ophoesten	☐	☐	☐	☐
**34.** Had je moeite met ademhalen	☐	☐	☐	☐
**35.** Had je buikpijn	☐	☐	☐	☐

*Kijk of **alle** vragen beantwoord zijn.*

HARTELIJK BEDANKT VOOR JE MEDEWERKING!

©2000 Quittner, Buu, Watrous en Davis

Opmerkingen:

©2000 Quittner, Buu, Watrous en Davis
