# Supplementary material for: How about your peers? Cystic fibrosis questionnaire data from healthy children and adolescents
Source: BMC Pediatr. 2011 Oct 11;11:86. doi: 10.1186/1471-2431-11-86 (PMC3198681; doi:10.1186/1471-2431-11-86)
Supplement: Additional file 2 — Cystic Fibrosis Questionnaire 12-13 Dutch version. Cystic Fibrosis Questionnaire for children aged 12-13 years (Dutch version). [file 1471-2431-11-86-S2.PDF]

CFQ CYSTIC FIBROSIS VRAGENLIJST

Kinderen 12 en 13 jaar (Format voor zelf-rapportage)

Deze vragen zijn voor kinderen als jij die Cystic Fibrosis hebben. Je antwoorden zullen ons helpen te begrijpen wat de ziekte betekent en hoe je behandelingen jou helpen. Daarom zal het beantwoorden van deze vragen jou en andere kinderen net als jij in de toekomst helpen.

Beantwoord alle vragen. Er zijn geen goede of foute antwoorden! Als je niet zeker weet hoe te antwoorden, kies dan het antwoord dat het dichtst bij jouw situatie in de buurt lijkt te komen.

Vul het antwoord in of kruis het hokje aan dat overeenkomt met je antwoord op deze vragen.

A. Wat is je geboortedatum?

Datum ☐☐ | ☐☐ | ☐☐
Dag Maand Jaar

B. Ben je een?

☐ Man ☐ Vrouw

C. Ben je in de **afgelopen 2 weken** normaal naar school geweest?

☐ Ja ☐ Nee

Zo nee, had dit iets te maken met je gezondheid?

☐ Ja ☐ Nee

D. In welke groep of klas zit je nu? (Als het zomer is, in welke groep of klas heb je het afgelopen jaar gezeten?)

☐ groep 7 basisschool
☐ groep 8 basisschool
☐ 1^{e} klas voortgezet onderwijs
☐ 2^{e} klas voortgezet onderwijs
☐ 3^{e} klas voortgezet onderwijs
☐ niet op school

In te vullen door testafnemer:

Datum ☐☐ | ☐☐ | ☐☐ (dag mnd jaar) Centrum ☐

1^{e} letters voornaam ☐ | ☐ | ☐ Patiënt # ☐☐☐☐☐☐☐☐
1^{e} letters achternaam ☐ | ☐ | ☐ Vanwege ☐

©2000 Quittner, Buu, Watrous en Davis

Kruis het hokje met je antwoord aan.

In de afgelopen **2 weken:**	Heel erg waar	Voor een groot deel waar	Een beetje waar	Helemaal niet waar
**1.** Kon je net zo snel lopen als andere kinderen	☐	☐	☐	☐
**2.** Kon je net zo snel traplopen als andere kinderen	☐	☐	☐	☐
**3.** Kon je rennen, springen en klimmen zoveel als je wilde	☐	☐	☐	☐
**4.** Kon je net zo snel en zo lang rennen als andere kinderen	☐	☐	☐	☐
**5.** Kon je meedoen met de sporten die je leuk vindt (bijvoorbeeld zwemmen, voetbal, dansen of andere)	☐	☐	☐	☐
**6.** Had je moeite zware dingen te dragen of te tillen zoals boeken, je schooltas of rugzak	☐	☐	☐	☐

Kruis het hokje met je antwoord aan.

En, in deze afgelopen **2 weken**, geef aan hoe vaak:	Altijd	Vaak	Soms	Nooit
**7.** Voelde je je moe	☐	☐	☐	☐
**8.** Voelde je je kwaad	☐	☐	☐	☐
**9.** Voelde je je mopperig	☐	☐	☐	☐
**10.** Maakte je je zorgen	☐	☐	☐	☐
**11.** Voelde je je verdrietig	☐	☐	☐	☐
**12.** Had je moeite in slaap te vallen	☐	☐	☐	☐
**13.** Had je enge dromen of nachtmerries	☐	☐	☐	☐
**14.** Voelde je je blij met jezelf	☐	☐	☐	☐

©2000 Quittner, Buu, Watrous en Davis

Kruis het hokje met je antwoord aan.

En, in deze **afgelopen 2 weken**, geef aan hoe vaak:

	Altijd	Vaak	Soms	Nooit
**15.** Had je moeite met eten	☐	☐	☐	☐
**16.** Moest je stoppen met spelen vanwege je behandelingen, zoals fysiotherapie, ademhalingsoefeningen, vernevelen, etc.	☐	☐	☐	☐
**17.** Moest je toch eten, terwijl je dat eigenlijk niet wilde	☐	☐	☐	☐

Kruis het hokje met je antwoord aan.

In de afgelopen **2 weken:**

	Heel erg waar	Voor een groot deel waar	Een beetje waar	Helemaal niet waar
**18.** Kon je al je behandelingen doen	☐	☐	☐	☐
**19.** Had je plezier in eten	☐	☐	☐	☐
**20.** Heb je veel met vriendjes / vriendinnetjes gespeeld	☐	☐	☐	☐
**21.** Ben je meer thuis gebleven dan je wilde	☐	☐	☐	☐
**22.** Voelde je je op je gemak om uit logeren te gaan	☐	☐	☐	☐
**23.** Voelde je je buitengesloten	☐	☐	☐	☐
**24.** Heb je vaak vriendjes/vriendinnetjes bij je thuis uitgenodigd	☐	☐	☐	☐
**25.** Ben je geplaagd door andere kinderen	☐	☐	☐	☐
**26.** Vond je het makkelijk om te praten over je ziekte met anderen (vrienden, klasgenoten, leraren, etc.)	☐	☐	☐	☐
**27.** Dacht je dat je te klein was	☐	☐	☐	☐
**28.** Dacht je dat je te mager was	☐	☐	☐	☐

Patiënt # ☐☐☐☐☐☐☐☐

©2000 Quittner, Buu, Watrous en Davis

In de afgelopen **2 weken:**	Heel erg waar	Voor een groot deel waar	Een beetje waar	Helemaal niet waar
**29.** Dacht je dat je lichamelijk anders was dan andere kinderen van jouw leeftijd	☐	☐	☐	☐
**30.** Vond je het vervelend om je behandelingen te doen	☐	☐	☐	☐

Kruis het hokje met je antwoord aan.

Laat ons weten hoe vaak in de afgelopen **2 weken:**	Altijd	Vaak	Soms	Nooit
**31.** Hoestte je overdag	☐	☐	☐	☐
**32.** Werd je 's nachts wakker omdat je hoestte	☐	☐	☐	☐
**33.** Moest je slijm ophoesten	☐	☐	☐	☐
**34.** Had je moeite met ademhalen	☐	☐	☐	☐
**35.** Had je buikpijn	☐	☐	☐	☐

Kijk of je alle vragen beantwoord hebt.

HARTELIJK BEDANKT VOOR JE MEDEWERKING!

©2000 Quittner, Buu, Watrous en Davis

Opmerkingen:

©2000 Quittner, Buu, Watrous en Davis
