# Supplementary material for: How about your peers? Cystic fibrosis questionnaire data from healthy children and adolescents
Source: BMC Pediatr. 2011 Oct 11;11:86. doi: 10.1186/1471-2431-11-86 (PMC3198681; doi:10.1186/1471-2431-11-86)
Supplement: Additional file 3 — Cystic Fibrosis Questionnaire 14+ Dutch version. Cystic Fibrosis Questionnaire for children aged 14 and/or older, and adults (Dutch version). [file 1471-2431-11-86-S3.PDF]

CFQ CYSTIC FIBROSIS VRAGENLIJST

Jongeren en volwassenen (patiënten van 14 jaar en ouder)

Het begrijpen van de invloed van je ziekte en behandelingen op je dagelijks leven kan je behandelteam helpen je gezondheid in de gaten te houden en je behandelingen bij te stellen. Daarom hebben we een vragenlijst ontwikkeld specifiek voor mensen met Cystic Fibrosis. We danken je voor je bereidwilligheid deze vragenlijst in te vullen.

Instructies: De volgende vragen gaan over je huidige gezondheidstoestand, zoals je deze ervaart. Deze informatie stelt ons in staat beter te begrijpen hoe jij jezelf in het dagelijks leven voelt.

Beantwoord alle vragen. Er zijn **geen** goede of foute antwoorden! Als je niet zeker weet hoe te antwoorden, kies dan het antwoord dat het dichtst bij je situatie in de buurt lijkt te komen.

Sectie I. Demografie

Vul de informatie in of kruis het hokje met je antwoord aan.

A. Wat is je geboortedatum?

Datum ☐☐ ☐☐ ☐☐
Dag Mnd Jaar

B. Ben je?

☐ Man ☐ Vrouw

C. Ben je in de **afgelopen 2 weken** normaal naar school/opleiding of werk geweest?

☐ Ja ☐ Nee

Zo nee, had dit iets te maken met je gezondheid?

☐ Ja ☐ Nee

D. Wat is je huidige burgerlijke staat?

- ☐ Ongehuwd/nooit gehuwd
- ☐ Gehuwd
- ☐ Weduwe/weduwnaar
- ☐ Gescheiden
- ☐ Hertrouwd
- ☐ Samenwonend met partner

E. Wat is de hoogste opleiding die je hebt voltooid.

- ☐ Basisschool
- ☐ VMBO (MAVO)
- ☐ LBO
- ☐ MBO
- ☐ HAVO
- ☐ VWO
- ☐ HBO
- ☐ Universiteit

F. Wat is je huidige werk/school situatie?

- ☐ Volg school/opleiding buitenshuis
- ☐ Volg cursussen thuis
- ☐ Werkzoekend
- ☐ Werk voltijds
- ☐ Werk deeltijds
- ☐ Doe voltijds huishoudelijk werk
- ☐ Volg geen school/opleiding of werk niet wegens gezondheidsproblemen
- ☐ Volg geen school/opleiding of werk niet wegens andere oorzaken

---

In te vullen door testafnemer:

Datum ☐☐ ☐☐ ☐☐ (dag mnd jaar) Centrum ☐ 1e letters voornaam ☐☐ 1e letters achternaam ☐☐ Patiënt # 0 ☐☐☐☐☐☐☐☐ Vanwege ☐

©2000 Quittner, Buu, Watrous en Davis

Sectie II. Kwaliteit van leven

Kruis het hokje van je keuze aan.

In welke mate heb jij ***gedurende de afgelopen 2 weken*** *moeite gehad om:*	Een heleboel moeite	Redelijk wat moeite	Een beetje moeite	Geen moeite
1. Flinke lichamelijke inspanningen te leveren, zoals rennen of sporten ..	☐	☐	☐	☐
2. Net zo snel te lopen als anderen	☐	☐	☐	☐
3. Zware dingen te dragen of te tillen, zoals boeken, een boodschappentas of schooltas	☐	☐	☐	☐
4. Eén trap op te lopen	☐	☐	☐	☐
5. Net zo snel als anderen de trap op te lopen	☐	☐	☐	☐

Geef aan hoe vaak jij je ***gedurende de afgelopen 2 weken****:*	Altijd	Vaak	Soms	Nooit
6. Goed voelde	☐	☐	☐	☐
7. Zorgen hebt gemaakt	☐	☐	☐	☐
8. Nutteloos voelde..	☐	☐	☐	☐
9. Vermoeid voelde	☐	☐	☐	☐
10. Energiek voelde..	☐	☐	☐	☐
11. Uitgeput voelde	☐	☐	☐	☐
12. Verdrietig voelde..	☐	☐	☐	☐

Omcirkel het nummer van je antwoord. Kies slechts één antwoord per vraag.

Denkend aan je gezondheidstoestand gedurende de afgelopen ***2 weken,*** *geef aan:*

13. De mate waarin je moeite had met lopen:
    1. Je kunt een lange tijd lopen zonder moe te worden
    2. Je kunt een lange tijd lopen, maar wordt moe
    3. Je kunt geen lange tijd lopen, omdat je snel moe wordt
    4. Je vermijdt lopen zo vaak mogelijk, omdat het te vermoeiend voor je is

14. Hoe je tegenover eten staat:
    1. Alleen al als je aan voedsel denkt, staat het je tegen
    2. Je eet nooit met plezier
    3. Je eet soms met plezier
    4. Je eet altijd met plezier

15. In welke mate je behandelingen je dagelijks leven moeilijker maken:
    1. Helemaal niet
    2. Enigszins
    3. Nogal
    4. Heel veel

©2000 Quittner, Buu, Watrous en Davis

16. Hoeveel tijd je dagelijks besteedt aan je behandelingen:
1. Heel veel tijd
2. Tamelijk veel tijd
3. Een beetje tijd
4. Nauwelijks tijd

17. Hoe lastig je het vindt om je behandeling (inclusief fysiotherapie, het innemen en inhaleren van medicijnen en dieetvoorschriften) elke dag uit te voeren:
1. Helemaal niet lastig
2. Een beetje lastig
3. Nogal lastig
4. Erg lastig

18. Hoe je vindt dat je gezondheid op dit moment is:
1. Uitstekend
2. Goed
3. Redelijk
4. Slecht

Kruis het hokje van je keuze aan.

*Denkend aan je gezondheidstoestand gedurende de afgelopen **2 weken**, geef aan in hoeverre elke uitspraak met betrekking tot jezelf waar of onwaar is:*	**Heel erg waar**	**Enigszins waar**	**Enigszins onwaar**	**Heel erg onwaar**
**19.** Ik heb moeite te herstellen na lichamelijke inspanning	☐	☐	☐	☐
**20.** Ik moet flinke lichamelijke inspanningen als rennen en sporten beperken	☐	☐	☐	☐
**21.** Ik moet mezelf dwingen om te eten	☐	☐	☐	☐
**22.** Ik ben meer aan huis gebonden dan ik wil	☐	☐	☐	☐
**23.** Ik vind het gemakkelijk om over mijn ziekte te praten met anderen	☐	☐	☐	☐
**24.** Ik vind mezelf te mager	☐	☐	☐	☐
**25.** Ik denk dat ik er anders uitzie dan mijn leeftijdgenoten	☐	☐	☐	☐
**26.** Ik voel me minder vanwege mijn uiterlijk	☐	☐	☐	☐
**27.** Mensen zijn bang dat mijn ziekte besmettelijk is	☐	☐	☐	☐
**28.** Ik ga vaak bij vrienden op bezoek	☐	☐	☐	☐
**29.** Ik denk dat mijn hoest anderen hindert	☐	☐	☐	☐
**30.** Ik voel me op mijn gemak als ik 's avonds uitga	☐	☐	☐	☐
**31.** Ik voel me vaak eenzaam	☐	☐	☐	☐
**32.** Ik voel me gezond	☐	☐	☐	☐
**33.** Het is lastig plannen te maken voor de toekomst (bijvoorbeeld gaan studeren, trouwen, carrière maken, enz.)	☐	☐	☐	☐
**34.** Ik leid een normaal leven	☐	☐	☐	☐

©2000 Quittner, Buu, Watrous en Davis

Sectie III. School, werk of dagelijkse bezigheden

De vragen 35 tot en met 38 hebben betrekking op je studie, je baan of je dagelijkse bezigheden.

35. In welke mate heb je in de afgelopen **2 weken** problemen ondervonden op school, op je werk of bij je dagelijkse bezigheden?
1. Je hebt geen moeite gehad om bij te blijven
2. Je bent er in geslaagd bij te blijven, maar het kostte moeite
3. Je hebt een achterstand opgelopen
4. Je hebt helemaal niets kunnen doen

36. Hoe vaak was je afwezig van school, werk, of niet in staat dagelijkse bezigheden af te maken in de afgelopen 2 weken vanwege je ziekte of behandelingen?

☐ Altijd ☐ Vaak ☐ Zelden ☐ Nooit

37. Hoe vaak wordt je gehinderd door CF bij het realiseren van doelstellingen op school, werk of op het persoonlijke vlak?

☐ Altijd ☐ Vaak ☐ Zelden ☐ Nooit

38. Hoe vaak belet CF je om naar buiten te gaan om een boodschap te doen, zoals gaan winkelen of naar de bank gaan?

☐ Altijd ☐ Vaak ☐ Zelden ☐ Nooit

Sectie IV. Problemen wat betreft symptomen

Kruis het hokje van je keuze aan.

*Geef aan hoe jij je de afgelopen **2 weken** hebt gevoeld.*	**Een heleboel**	**Redelijk veel**	**Een beetje**	**Helemaal niet**
**39.** Heb je moeite gehad om aan te komen in gewicht?	☐	☐	☐	☐
**40.** Heb je het benauwd gehad?	☐	☐	☐	☐
**41.** Heb je overdag gehoest?	☐	☐	☐	☐
**42.** Heb je sputum opgehoest	☐	☐	☐	☐ **Ga naar vraag 44**

43. Je sputum was meestal: ☐ Helder ☐ Helder tot geel ☐ Gelig-groen ☐ Groen met sporen bloed ☐ Weet ik niet

*Hoe vaak gedurende de afgelopen **2 weken:***	**Altijd**	**Vaak**	**Soms**	**Nooit**
**44.** Had je een piepende ademhaling?	☐	☐	☐	☐
**45.** Had je moeite met ademhalen?	☐	☐	☐	☐
**46.** Werd je 's nachts wakker omdat je hoestte?	☐	☐	☐	☐
**47.** Heb je last van winderigheid gehad?	☐	☐	☐	☐
**48.** Heb je diarree gehad?	☐	☐	☐	☐
**49.** Heb je buikpijn gehad?	☐	☐	☐	☐
**50.** Heb je problemen gehad met eten?	☐	☐	☐	☐

Kijk of je alle vragen beantwoord hebt.

©2000 Quittner, Buu, Watrous en Davis

HARTELIJK BEDANKT VOOR JE MEDEWERKING!

©2000 Quittner, Buu, Watrous en Davis
